# Improving complex health systems and lived environments for maternal and perinatal well-being in urban sub-Saharan Africa: the UrbanBirth Collective

**DOI:** 10.7189/jogh.15.03009

**Published:** 2025-01-23

**Authors:** UrbanBirth Collective, Mena Komi Agbodjavou, Christian Agossou, Anteneh Asefa, Elias Martinien Avahoundje, Catherine Birabwa, Janvier Kubuya Bonane, Christelle Boyi Hounsou, Sékou Niouma Camara, Facely Camara, Rachel Cassidy, Bintou Condé, Alexandre Delamou, Justin Lewis Denakpo, Souleymane Diakité, Nafissatou Dioubaté, Stefanie Dens, Jean-Paul Dossou, Fassou Mathias Grovogui, Yiséché SC Hounmenou, Tabitha Ilunga, Dieney Fadima Kaba, Amani Idris Kikula, Pépé Kpogomou, Niclette Lakula, Thierry Lawale, Peter M Macharia, Abdulu Mahuridi, Francoise Malonga, Hawa Manet, Tamba Mina Millimouno, Marie Alice Mosuse, Abel Ntambue Mukengeshayi, Nathalie Mulongo, Angele Musau, Debaïf Mutombo, Albert Tambwe-A-Nkoy Mwembo, Rehema Ouko, Claudia Nieto Sanchez, Mamoudou Sangaré, Aline Semaan, Manuela Straneo

## Abstract

While maternal mortality decreased during the Millennium Development Goals era, it remains unacceptably high, with stagnation in reductions possible due to shocks such as COVID-19. Most women in low- and middle-income countries already receive antenatal care and over half give birth in health facilities. In cities, use of health facilities for childbirth is near universal (>90%). Cities present complex challenges in ensuring pregnant women receive equitable, high-quality care. The UrbanBirth Collective is a portfolio of projects in sub-Saharan African cities seeking to address an important knowledge gap: how to adapt urban healthcare systems and lived environments to improve maternal and perinatal well-being? Its key focus is care during labour, childbirth, and the early postnatal period, when most poor maternal and perinatal outcomes occur. Our starting projects focus on harnessing open source data to examine and compare cities on the continent, including in-depth case studies of three cities: Grand Conakry (Guinea), Grand Nokoué metropolitan area (Benin), and Lubumbashi (Democratic Republic of the Congo), where we will capture and analyse three main dimensions of the dynamics: maternal health service provision; maternal healthcare use by women; and the complex, nonlinear interactions between the provision and use of care within the spatial, social, and political ecosystem of a city. By comparing these three cities, we shall propose a generalisable model which can be validated and applied in other cities in sub-Saharan Africa. The growth of cities demands increasing attention on future-proofing them with the capacity to develop, implement, and continuously adapt a coherent strategy for the provision of equitable maternal and newborn care. Our ambition is to contribute to reaching zero preventable maternal deaths in cities. To achieve these goals through understanding specific contexts and facilitating the adoption and application of research findings and recommendations, we will collaborate closely with local stakeholders, including healthcare workers, community leaders, and policymakers.

Maternal mortality reduced by 34% between 2000 and 2020, but this progress was insufficient to meet the Millennium Development Goal 5 [1]. With the current global maternal mortality ratio at 223 per 100 000 live births, we are also not on track to meet the 2030 Sustainable Development Goal (SDG) target of less than 70. Across the world, levels of maternal mortality vary from <6 per 100 000 live births (*e.g.* in Belgium and Sweden) to over 1000 (Chad and South Sudan). This 200-fold difference between levels is one of the most shocking, unfair, and damaging social phenomena of our time [2]. Around five million stillbirths and neonatal (jointly referred to as perinatal) deaths annually reflect the same challenges for equitable health systems [3,4]. This burden is concentrated in sub-Saharan Africa (SSA), where 42% of stillbirths occurred in 2019, compared to 2% in Europe, Northern America, Australia, and New Zealand combined [5]. Similarly, the risk of death in the first month of life is 11 times higher in SSA compared to Australia and New Zealand [6].

Most maternal and perinatal deaths result from complications occurring within a short time period between the onset of labour and the first days after birth. Identifying and treating such complications requires timely access (within 1–2 hours) to skilled clinical care, usually only found in well-capacitated health facilities [7,8]. However, preventable maternal and perinatal mortality is not just a result of lack of knowledge and resources. It derives from a sub-optimally functioning ‘ecosystem’, where the conditions of life-saving care provision, women’s health-seeking behaviours and financial means, and the landscape of how women reach health facilities (location of hospitals vs population concentration, referral systems, roads [9,10], traffic, *etc.*) interact. It is this complex and persistent problem [11] – a result of human decisions, organisation, and structures – which is the root cause of preventable maternal and perinatal mortality and morbidity. Historically, most research on accessibility of maternal healthcare has been focussed on rural areas due to the role of geographic distance, socioeconomic disadvantage, insufficient roads and transport options, and lower density of health facilities [12,13]. However, two-thirds of the world’s population will live in urban areas by 2050 and nearly 90% of these additional 2.5 billion urban residents will be concentrated in Africa and Asia [14]. In most low-resource settings, urban dwellers might have better access to care than their rural counterparts due to the urban bias in healthcare provision. However, failure to understand and address the complex challenges of timely access to high-quality care, particularly among vulnerable groups in urban settings, is a major obstacle to achieving the SDGs.

## MATERNAL AND PERINATAL HEALTH WITHIN URBAN HEALTH SYSTEMS

Two important recent paradigm shifts have gained recognition within the fields of maternal health and health systems: the increase in health-seeking and the complexity of urban health systems. However, we have not yet developed an effective conceptual and methodological approach to tackle them jointly, particularly in urban settings.

The first shift is an increase in health-seeking [15]. Unlike at any time in human history, most women in the world use modern medical care in health facilities during pregnancy and childbirth. Between 2001 and 2021, the percentage of women who gave birth in a health facility rose from 51% to 80% globally [16]. This increase is thought to have contributed to the decline in maternal and perinatal mortality [17]. Further, the uptake of maternal care is now near universal in large cities, even in low-resource settings. A recent study on 22 large cities in Africa showed that the median uses of facility-based antenatal and childbirth care were 98% and 95%, respectively [18]. This would suggest that the problem is not that women are reluctant to seek care; it is rather that many women seek care in facilities which are not able to treat obstetric emergencies (*e.g.* caesarean section, blood transfusion, newborn resuscitation) or provide harmful care and delay effective onward referral. The consequences of this are clear – more maternal deaths are attributable to poor quality of care than to lack of access to care [19]. Yet, access to such high-quality obstetric emergency care remains a concern in urban areas in SSA.

Beyond interventions aiming to address issues of sub-optimal quality of facility-based care [20–22], broader research in urban settings has produced important work to understand linkages between facilities – the so-called networks of care [23] and referral networks [24,25]. Progress has also been achieved in implementation research attempting to decongest referral hospitals (redistribute the number of women seeking childbirth care) by strengthening childbirth care provision to district-level facilities and health centres, such as in Dar es Salaam [26]. A new direction includes identifying urban geographic areas with suboptimal healthcare provision by studying travel times for women in obstetric emergencies based on big data, as was recently applied in a study on maternal care across 15 large cities in Nigeria [27–31].

This leads us to the second shift, which is the complexity of urban health systems [32–34]. Most women now live in rapidly urbanising areas [35,36]. In rural areas, hospital catchment areas are non-overlapping, and management of referrals is simple and linear if there are sufficient resources and communication [37,38]. Cities, on the other hand, produce interactions between population, health conditions, socioeconomic circumstances, physical environment, political governance, climate and environmental factors, health-seeking behaviours and provision of care which are much more complex than anything we have tackled on a system level in maternal health [39,40]. This complexity has a real impact on human health: adult mortality differentials between urban and rural areas have narrowed or even reversed [41,42]. Newborn mortality in core urban areas in Tanzania is two-fold higher compared to rural areas, and this pattern might be unfolding in other large countries, including Ghana, Uganda, and Kenya [43]. However, we do not know which factors contribute to this difference and how, limiting our ability to take effective action. Additionally, urban environments influence the ways in which girls and women grow, live, work, and seek healthcare services, and consequently their physical and mental health. Efforts to improve maternal and perinatal health in cities need to recognise that women navigate cities differently to men [44,45]. This means gender-sensitive design of urban health systems is critical to reducing maternal and perinatal mortality [46].

Urban health systems are infrequently studied [47] and struggle to meet the needs of women. Some of the challenges co-occurring in cities include clustering of urban poverty and marginalisation of migrants from the health system [48]; too many health facilities providing low levels of care, including a large and poorly regulated private sector contributing to overmedicalisation [49] and high out-of-pocket expenditure [50]; suboptimal quality of care in health facilities [51,52]; relatively short travel distances to health facilities obfuscating long travel times [18] – *e.g.* traffic resulting in delays; inability to travel at night due to insecurity [53–56]; lack of trust in facilities resulting in by-passing of nearest facility (even in emergency) and crowding in hospitals [26,53]; ineffective and inefficient referral systems [57] and facility communication channels, alongside scarcity of functioning ambulances [58,59]; and incomplete or poor quality routine data to inform decision-makers [31]. Other, highly-specific local factors exist in each city; many are not yet identified or well described [60]. These include environmental factors (*e.g.* air pollution, effect of heat), but also broader sociopolitical issues critical to health systems decision-making, such as what ‘urbanicity’ means [61], whether and how the administrative boundaries of a city align with the growth of human settlements [62], and whether the concept of health catchment areas based on geography is useful in large cities at all [37,38].

## CHALLENGES IN UNDERSTANDING THE COMPLEX PROBLEM

Clinical causes of maternal and perinatal deaths are well known. If women are at the right place (a well-functioning and capable health facility) at the right time (before or within a few hours of developing an obstetric complication), most of them and their babies survive. However, in complex urban health systems, we lack methods and tools to identify the most critical problems blocking further reductions in preventable mortality.

Several challenges have prevented the kind of large-scale research which is required to elucidate the nature of the maternal health dynamics in cities and inform context-specific actions. The most pertinent of these challenges are the framing of research questions and data availability to answer questions related to complexity. More specifically, narrowly framed research questions which are inadequate in urban contexts are the first challenge. In this sense, a lack of a ‘big picture’ thinking has meant that research has focussed on one narrow issue, which has resulted in fragmented, incomplete, and sometimes incorrect answers to the question on which are the key barriers to improving maternal well-being and survival in cities. Examples include initiatives that promote hospital-based births as the only option to improve acces to quality of care [63–65]; a health system redesign concept that does not fully incorporate the challenges of limited resources [66]; the risk of overmedicalisation and increase in disrespectful care [67–70]; and the complex sociocultural dynamics driving women’s choices of place of birth [71–74] in SSA. Another set of studies has identified barriers to accessibility of care at the appropriate level. Some focussed on geographic accessibility of care in cities [30,55,75,76], others considered the functionality of intra-facility referral networks (ambulance availability, communications, *etc.*) [57,77–79] or improving the functionality of several linked facilities which constitute a ‘network of care’ (*e.g.* in Manila) [23,80–82], while some investigated financial barriers to women’s pathways through care such as informal user fees and lack of financial decision-making [83]. These studies are extremely valuable in exposing the extent of these challenges and developing innovative methods to measure the burden. However, because they do not measure the influence of a wide range of factors, they are unable to construct and interrogate the complex reality of the city ecosystem and position their findings among a larger set of social, political, and health system realities and competing political and health system priorities. In-depth, context-specific research on the challenges to improve maternal and perinatal health in cities does exist, however, with successful examples from Dar es Salaam (Tanzania) [26,84] and Rotterdam (the Netherlands) [85–87]. This research shows that a comprehensive nature of inquiry into issues influencing maternal and perinatal survival and well-being is possible and impactful. Yet, case studies of single cities do not allow for rigorous multi-city comparison and pattern recognition which could inform generalisation across cities.

Such multi-city analysis is dependent on tackling the second challenge, which is the unavailability of accurate and comparable data about the various elements of maternal healthcare dynamics in cities [88]. While open-access data sets of health facilities and their location in SSA are available [89], they suffer from errors of omission (private sector facilities, which provide 20–40% of childbirth care, are excluded or out-of-date), lack attributes of services offered, or are misclassified (true functioning status in terms of provision of emergency obstetric care is different from the official designation). Some data sources which can help characterise the choices and pathways of women through the healthcare system exist: these include household surveys such as the Demographic and Health Surveys (DHS), which are standardised household surveys conducted in many countries every five years. The questionnaires ask women to self-report about where they sought care during recent pregnancies, but exclude key populations (women who died from a maternal cause of death), do not capture complications during pregnancy/birth (women’s report of obstetric complications has low specificity), and do not ask questions about preferences and reasons for seeking care or not. Furthermore, the sample sizes of households surveyed in cities are commonly not sufficient to produce city- or conurbation-level estimates for key indicators. Next, data generated by the routine health information systems (District Health Information System 2 (DHIS2) or *Système national d'information sanitaire* (SNIS) in French) capture basic aggregate monthly indicators of care use reported by health facilities to regional and national authorities (*e.g.* number of births, number of caesarean sections, number of newborn deaths). While DHIS2 data are available on a continuous basis, they have several limitations, including incomplete reporting where several facilities are not captured, omission of certain key indicators such as maternal deaths and stillbirths [90], and the exclusive capture of women who sought care in facilities and determination of denominator for different indicators. Although both DHS and DHIS2 data are useful for health system and population health monitoring, they do not capture individual women’s pathways to and through care [91] or link them to health outcomes. Last, available data are not linked for purposes of understanding the dynamics: for example, we know where women live, but we do not know which health facilities they use and why; we know how many obstetric referrals a given hospital receives, but not where such cases originated, why they were referred, how long they travelled, and what the outcomes were.

## TOWARD A SOLUTION

The UrbanBirth Collective is a partnership of research institutions working together with stakeholders toward context-specific understanding of problems and solutions in SSA cities. Our ambition is to reach zero preventable maternal and perinatal deaths in cities. We aim to accomplish this by addressing an important knowledge gap: how to adapt urban healthcare systems and lived environments to most effectively improve on maternal and perinatal survival and well-being. Four research organisations founded this initiative in 2024: Institute of Tropical Medicine, Antwerp, Belgium; *L'Ecole de Santé Publique de l'Université de Lubumbashi* (ESP/UNILU) in the Democratic Republic of the Congo; *Centre d’Excellence Africain pour la Prévention et le Contrôle des Maladies Transmissibles* (CEA-PCMT) in Guinea, and *Centre de Recherche en Reproduction Humaine et en Démographie* (Cerrhud) in Benin.

**Figure 1:**
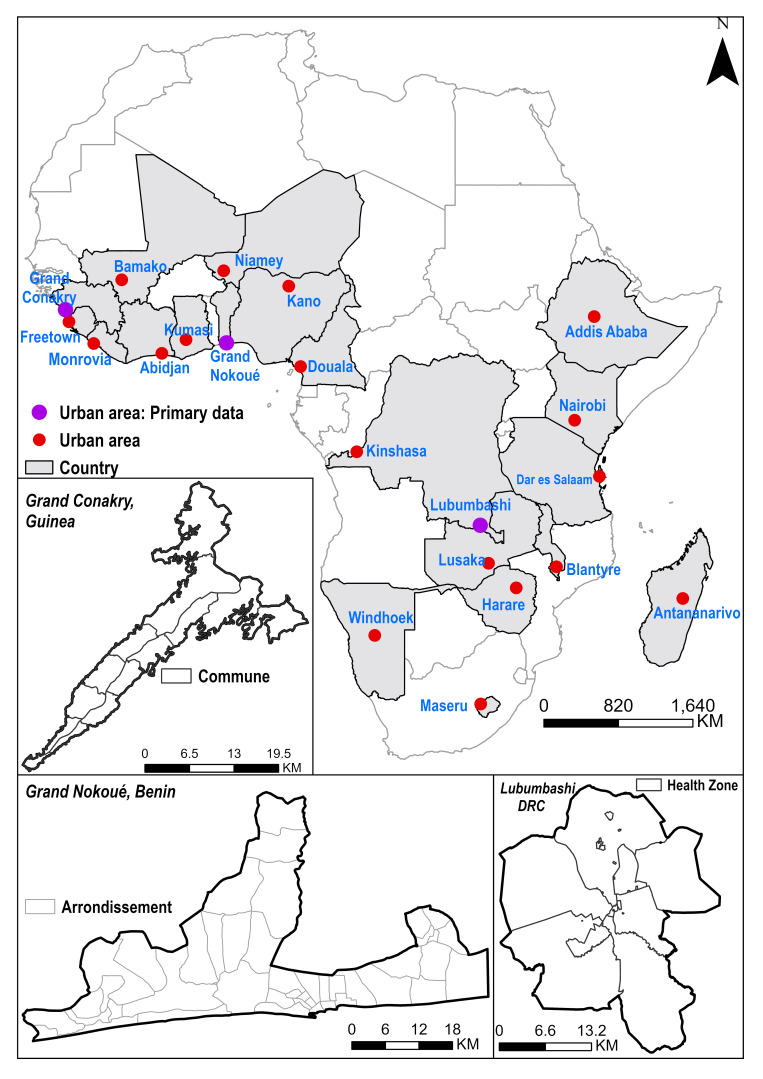
Map showing cities in sub-Saharan Africa for which primary (purple) and secondary (red) data are being analysed within research projects of the UrbanBirth Collective.

Our initial focus is two-fold. First, we want to use secondary data (satellite imagery, national household surveys, and sociodemographic data) within an advanced geospatial modelling framework to define a novel maternal health vulnerability index over the last three decades across 21 urban areas in SSA (**Figure 1**; Table S1 in the **Online Supplementary Document**). The index encapsulates factors (obstacles) that make women vulnerable: environmental, sociodemographic, disease burden, poor access to and low-quality healthcare, suboptimal health-seeking, and dysfunctional health systems. The second focus area encompasses specific understanding of three cities based on primary data, complemented by comparative work to identify commonalities across the three cities: Grand Conakry in Guinea, Lubumbashi in the Democratic Republic of Congo, and the Grand Nokoué Metropolitan area in Benin (**Table 1**). Through these two lenses and comparative work, we aim to develop a comprehensive system dynamics model [92] of the ecosystem of maternal healthcare provision in urban areas which leads to key maternal and perinatal health outcomes. We will also test the generalisability of this mathematical model for use in similar urban settings in SSA.

**Table 1:** Description of main cities of focus*

Country	Benin		DRC	Guinea	Range across other 17 countries
Country population (in millions, 2024)	14.5	109.3		14.8	2.3–232.7
Percentage of population living in urban areas (2022)	50	43		43	17–59
Maternal mortality ratio per 100 000 live births (2020)	523	547		553	135–1047
Stillbirth rate per 1000 births (2021)	20	28		23	14–28
Neonatal mortality rate per 1000 live births (2022)	29	26		31	19–35
Total fertility rate (2023)	4.6	6.1		4.2	2.7–6.1
**City**	**Grand Nokoué metropolitan area**		**Lubumbashi**	**Grand Conakry**	**Range across other 18 cities**
City population (in millions, 2023)	2.9		2.8	2.1	0.5–16.3
Name of area/region which includes city and year of estimate	Cotonou (2018)		Haut Katanga (2014)	Conakry (2018)	
*Percentage of all births in health facilities*	99.0		94	90	20–99
*Percentage of births by caesarean section*	15		11	6	1.3–28
*Neonatal mortality rate per 1000 live births*	24		35	24	12–49

*Source of data for each indicator and list of cities and their characteristics are available in Table S1 in the **Online Supplementary Document**.

At the core of our Collective are four key values guiding our thinking and ways of working. Starting with open science, we strive to make all data open-access, including study protocols and resulting data, within data protection and safety requirements in place. We aim to publish in open-access journals and repositories, and to disseminate our findings to various audiences (*e.g.* communities, journalists, health service providers and managers, policymakers, and academics) using appropriate communication channels and media. In alignment, our second value is close engagement and collaboration with stakeholders from the conception of the study, to ensure interest and relevance of the work within each local context and to enable the uptake of the findings (translation of the results into policy and practice). We plan to hold repeat stakeholder engagement meetings in each of the three cities, through which we will share our progress and receive feedback to tailor the project to meet stakeholder needs and interests. The nature of the stakeholders underlines our third value, which is multisectoral collaboration. Urban maternal and perinatal health presents itself as a complex problem, which warrants engagement with experts from various fields, including policy and decision makers, researchers, academics, epidemiologists, clinicians, experts in urbanisation, city planning and design, environmental specialists, *etc*. Fourth, this project offers several opportunities for learning and capacity strengthening, which we plan to leverage by working with early-career PhD and postdoctoral researchers, offering a platform for knowledge exchange and cross-learning.

As a Collective, we have a common agenda and platform for exchange on developing an understanding of how various factors and their interactions result in maternal and perinatal mortality in cities, given a set of health system resources, population characteristics, and interactions between these factors and the urban environment. This will enable local and national stakeholders to rapidly assess and compare the effects of various actions to improve maternal and perinatal health outcomes in cities, and more broadly, inform how to structure urban health systems to deliver better care accessibility and quality. To correctly describe and understand the nonlinear, dynamic relationships between various elements of the urban maternal health ‘ecosystem’, we developed a conceptual approach and will use extensive depth and breadth of data. Our focus is on these key parameters: availability and capability of health facilities, including quality of care (element 1); women’s decisions and health-seeking behaviours (element 2); the physical, social, and political environment in which people and institutions operate in cities (element 3); and the ways in which changes in elements 1, 2, and 3 interact and operate to influence maternal and perinatal mortality.

**Figure 2:**
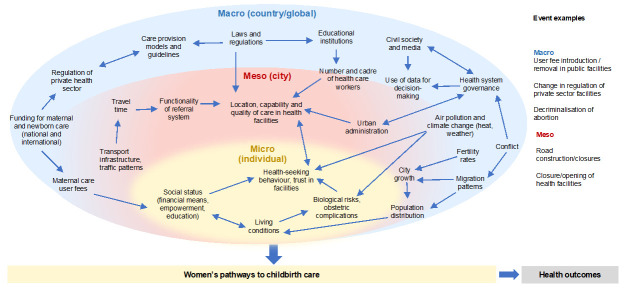
Conceptual framework showing levels and elements of urban maternal healthcare ecosystem [94,95].

We use the ecological conceptual framework [93] in capturing these interacting elements on micro (individual women and their families), meso (urban environment), and macro (national and international) levels (**Figure 2**), as it allows for the identification and assessment of salient elements of the urban maternal health ecosystem and the effect of their interactions on the pathways to childbirth care, and finally, on health outcomes. Micro-level elements allow for the understanding of how women make choices about when and where to seek childbirth care, considering their socioeconomic background, risk perception, and care options. Meso-level elements relate to the city-level geographic and sociopolitical environment, such as the distribution of population, road infrastructure, location and quality of care in health facilities, referral system, and the political structures in charge of the health system [96]. Macro-level captures elements of society, health systems, health worker education [97], policy, and the lived environment at the national and global level. These are incorporated here because they inform the urban health ecosystem.

The UrbanBirth Collective research projects begin with collection, collation, and analysis of secondary data on household surveys, satellite imagery, urbanisation grids, within city boundaries, location of informal settlements, conflict zones, population distribution, relative wealth index, precipitation, temperature, health facilities, road infrastructure, travel speeds within cities, and others. Additional information on care provision will be gathered through aggregate routine data collected by health authorities in the three cities. Primary data collection includes surveys of health facilities providing maternal and newborn care in the cities capturing their location, capability, functionality and interconnectedness within referral networks. We will also gather information on patterns of women’s use of care collected through qualitative interviews. Understanding how the ‘ecosystem’ operates and produces outcomes requires a close up tracking of how it operates.

In Conakry and Lubumbashi, prospective data will be collected about events in the cities on the three levels of the conceptual framework (**Figure 2**). The data on these events will be analysed together with key maternal and perinatal health indicators (number of births, caesarean sections, referrals, and maternal/perinatal deaths) from all large health facilities to reveal interdependencies and nonlinear relations affecting maternal and perinatal mortality. When mathematically expressed, this in-depth understanding of cause-and-effect on a spatial and health system level will inform effective action that improves maternal and perinatal health outcomes. The comparison of results across cities will allow us to identify generalisable patterns, while the identified links will be leveraged to inform appropriate interventions to be designed in collaboration with key stakeholders in each city. The interventions can then be used beyond the three cities as the basis of understanding maternal healthcare provisions and dynamics in other cities across SSA, and the work will inform the required amount and specificities of data to enable establishing similar initiatives elsewhere.

## CONCLUSIONS

In 2030, over 60 million births will occur in urban areas in low- and middle-income countries [98]. Now is the time to invest in ensuring good quality maternal and newborn care and tackling high levels of inequality in healthcare provision and access in urban areas. The systems and architecture of ‘smart cities’ [99] of the future is being laid now – maternal and perinatal health cannot be left behind in this process. The ambition of the UrbanBirth Collective is to develop and lead this field at the interface of health systems, women’s needs, and urban environments. This will happen by answering questions such as:

Which patterns of the maternal healthcare ecosystem in SSA cities are generalisable?
Which patterns are context-specific, and why?
To what extent does a model based on generalisable patterns accurately predict maternal and perinatal mortality in a city where less granular data are available?
Could the effects of interventions on reducing maternal and perinatal mortality be accurately predicted using a model?
More generally, what are minimum data requirements to monitor the state of maternal and perinatal health on the scale of a city?

Our philosophy is to engage and collaborate with multisectoral stakeholders while respecting the primacy of local ownership of data and research findings across the three main cities throughout the lifetime of these different projects. We will disseminate the outputs using a phased approach. For example, when we map all health facilities and the corresponding subnational boundaries (health area and health zones) in a city, we will validate and disseminate these findings. We will be practising open science. Our research protocols and generated data sets are or will be open-access, including our manuscripts. We will make outputs more accessible to non-research communities by sharing our experiences and outputs *via* blog posts [100] and social media. The Collective is also being used as a basis of training and cross-learning between institutions and researchers, whereby the learning entails completion of PhDs, exchange between postdoctoral scientists and other early-career researchers.

Finally, the Collective’s contribution extends beyond urban maternal health. Findings on coordination of emergency obstetric care, women’s preferences of person-centred care, and the interactions of these two dimensions can be used to inform other fields of healthcare in cities, such as non-communicable diseases, emergency care, and general practice. A broad contribution lies in generating knowledge that informs health system readiness and response to future events and shocks, including epidemics, natural disasters, population displacement and climate change. Recommendations for this research have the potential to cross various disciplines, including urban design and governance.

## Additional material


Online Supplementary Document
