# Supplementary material for: Improving complex health systems and lived environments for maternal and perinatal well-being in urban sub-Saharan Africa: the UrbanBirth Collective
Source: J Glob Health. 2025 Jan 23;15:03009. doi: 10.7189/jogh.15.03009 (PMC11755202; doi:10.7189/jogh.15.03009)
Supplement: Online Supplementary Document [file jogh-15-03009-s001.pdf]

**Supplement to: UrbanBirth Collective. Improving complex health systems and lived environments for maternal and perinatal well-being in urban sub-Saharan Africa: the UrbanBirth Collective. J Glob Health. 2025;15:03009.**

## Table S1. Characteristics of 21 cities in UrbanBirth Collective projects

| Country | Benin | DRC | Guinea | Cameroon | Niger | Ghana | Côte d'Ivoire |
|---|---|---|---|---|---|---|---|
| Country population (in million, 2024) | 14,5 | 109,3 | 14,8 | 29,1 | 27,0 | 34,4 | 31,9 |
| % of population living in urban areas (2023) | 50% | 43% | 43% | 67% | 23% | 59% | 59% |
| Maternal mortality ratio per 100,000 live births (2020) | 523 | 547 | 553 | 438 | 441 | 263 | 480 |
| Stillbirth rate per 1,000 births (2021) | 20 | 28 | 23 | 19 | 21 | 21 | 22 |
| Neonatal mortality rate per 1,000 live births (2022) | 29 | 26 | 31 | 26 | 34 | 21 | 29 |
| Total fertility rate (2023) | 4,6 | 6,1 | 4,2 | 4,3 | 6,1 | 3,4 | 4,3 |
| **City** | **Grand Nokoué metropolitan area** | **Lubumbashi** | **Grand Conakry** | **Douala** | **Niamey** | **Kumasi** | **Abidjan** |
| City population (mil, 2023) | 2,9 | 2,8 | 2,1 | 4,1 | 1,4 | 3,8 | 5,7 |
| City area (km$^2$) | 1,284 | 747 | 450 | 923 | 297 | 214 | 2119 |
| Name of area/region which includes city and year of estimate | Cotonou (2018) | Haut Katanga (2014) | Conakry (2018) | Douala province (2018) | Regions of Niamey (2012) | Region of Ashanti (2022) | Districts of Abidjan (2021) |
| % of all births in health facilities | 99,0 | 94,0 | 90,0 | 98,3 | 91,0 | 92,4 | 94,5 |
| Caesarean section rate in % | 15 | 11 | 6 | 12 | 9 | 23 | 17 |
| Neonatal mortality rate per 1,000 live births* | 24 | 35 | 24 | 18 | 22 | 23 | 31 |

| Country | Mali | Liberia | Sierra Leone | Ethiopia | Kenya | Zambia | Malawi |
|---|---|---|---|---|---|---|---|
| Country population (in million, 2024) | 24,5 | 5,6 | 8,6 | 132,1 | 56,4 | 21,3 | 21,7 |
| % of population living in urban areas (2023) | 45% | 56% | 46% | 22% | 29% | 44% | 25% |
| Maternal mortality ratio per 100,000 live births (2020) | 440 | 652 | 443 | 267 | 530 | 135 | 381 |
| Stillbirth rate per 1,000 births (2021) | 23 | 23 | 23 | 21 | 19 | 14 | 16 |
| Neonatal mortality rate per 1,000 live births (2022) | 33 | 30 | 31 | 27 | 20 | 24 | 19 |
| Total fertility rate (2023) | 5,6 | 4,0 | 3,8 | 4,0 | 3,2 | 4,1 | 3,6 |
| **City** | **Bamako** | **Monrovia** | **Freetown** | **Addis Ababa** | **Nairobi** | **Lusaka** | **Blantyre** |
| City population (mil, 2023) | 2,9 | 1,7 | 1,3 | 5,5 | 5,3 | 3,2 | 1,0 |
| City area (km$^2$) | 267 | 194 | 82 | 527 | 704 | 360 | 228 |
| Name of area/region which includes city and year of estimate | Region of Bamako (2018) | Monrovia (2020) | Western Urban Districts (2019) | State of Addis Ababa (2019) | Counties of Nairobi (2022) | Province of Lusaka (2018) | Blantyre City (2016) |
| % of all births in health facilities | 97,4 | 82,4 | 94,6 | 96,5 | 99,4 | 92,2 | 93,2 |
| Caesarean section rate in % | 5 | 8 | 10 | 24 | 28 | 9 | 11 |
| Neonatal mortality rate per 1,000 live births* | 20 | 43 | 37 | 17 | 20 | 27 | 28 |

| Country | Zimbabwe | Lesotho | Namibia | Nigeria | Tanzania | DRC | Madagascar |
|---|---|---|---|---|---|---|---|
| Country population (in million, 2024) | 16,6 | 2,3 | 3,0 | 232,7 | 68,6 | 109,3 | 32,0 |
| % of population living in urban areas (2023) | 46% | 36% | 46% | 58% | 34% | 43% | 38% |
| Maternal mortality ratio per 100,000 live births (2020) | 357 | 566 | 215 | 1047 | 238 | 547 | 392 |
| Stillbirth rate per 1,000 births (2021) | 19 | 27 | 17 | 22 | 18 | 28 | 18 |
| Neonatal mortality rate per 1,000 live births (2022) | 24 | 35 | 19 | 34 | 20 | 26 | 24 |
| Total fertility rate (2023) | 3,7 | 2,7 | 3,2 | 4,5 | 4,6 | 6,1 | 4,0 |
| **City** | **Harare** | **Maseru** | **Windhoek** | **Kano** | **Dar es Salam** | **Kinshasa** | **Antananarivo** |
| City population (mil, 2023) | 1,6 | 0,6 | 0,5 | 4,3 | 7,8 | 16,3 | 3,9 |
| City area (km$^2$) | 960 | 138 | 5133 | 20131 (Kano state) | 1590 | 9 965 | 86 |
| Name of area/region which includes city and year of estimate | Province of Harare Chitungwiza (2015) | Districts of Maseru (2014) | Regions of Khomas (2013) | Kano state (2018) | Region of Dar es Salaam (2022) | Province of Kinshasa (2014) | Antananarivo capital (2021) |
| % of all births in health facilities | 94,1 | 82,4 | 95,2 | 20 | >99 | 98,3 | 68 |
| Caesarean section rate in % | 10 | 11 | 26 | 1 | 26 | 7 | 10 |
| Neonatal mortality rate per 1,000 live births* | 23 | 31 | 12 | 37 | 49 | 16 | 27 |

| Country | Source |
| --- | --- |
| Country population (in million, 2024) | https://www.worldometers.info/world-population/population-by-country/ |
| % of population living in urban areas (2022) | Our Word in Data |
| Maternal mortality ratio per 100,000 live births (2020) | https://iris.who.int/bitstream/handle/10665/372258/9789240069312-fre.pdf?sequence=1&isAllowed=y |
| Stillbirth rate per 1,000 births (2021) | https://childmortality.org/all-cause-mortality/data/download |
| Neonatal mortality rate per 1,000 live births (2022) | https://childmortality.org/all-cause-mortality/data/download |
| Total fertility rate (2023) | https://ourworldindata.org/fertility-rate |
| **City** | |
| City population (mil, 2023) | United Nations Department of Economic and Social Affairs Population Dynamics |
| City area (km$^2$) | Source for each city differs |
| Name of area/region which includes city and year of estimate | |
| % of all births in health facilities | DHS StatCompiler |
| Caesarean section rate in % | DHS StatCompiler |
| Neonatal mortality rate per 1,000 live births | DHS StatCompiler |
